# Identification of a New Mullet Species Complex Based on an Integrative Molecular and Cytogenetic Investigation of *Mugil hospes* (Mugilidae: Mugiliformes)

**DOI:** 10.3389/fgene.2018.00017

**Published:** 2018-02-05

**Authors:** Mauro Nirchio, Fabilene G. Paim, Valentina Milana, Anna R. Rossi, Claudio Oliveira

**Affiliations:** ^1^Facultad de Ciencias Agropecuarias, Universidad Técnica de Machala, Machala, Ecuador; ^2^Departamento de Morfologia, Instituto de Biociências, Universidade Estadual Paulista “Júlio de Mesquita Filho”, São Paulo, Brazil; ^3^Dipartimento di Biologia e Biotecnologie “C. Darwin”, Sapienza Università di Roma, Rome, Italy

**Keywords:** fish cytogenetics, fish molecular phylogeny, COI, chromosomal evolution, FISH, Mugilidae

## Abstract

Mullets are very common fishes included in the family Mugilidae, (Mugiliformes), which are characterized by both a remarkably uniform external morphology and internal anatomy. Recently, within this family, different species complexes were molecularly identified within *Mugil*, a genus which is characterized by lineages that sometimes show very different karyotypes. Here we report the results of cytogenetic and molecular analyses conducted on *Mugil hospes*, commonly known as the hospe mullet, from Ecuador. The study aims to verify whether the original described species from the Pacific Ocean corresponds to that identified in the Atlantic Ocean, and to identify species-specific chromosome markers that can add new comparative data about Mugilidae karyotype evolution. The karyotype of *M. hospes* from Ecuador is composed of 48 acrocentric chromosomes and shows two active nucleolar organizer regions (NORs). *In situ* hybridization, using different types of repetitive sequences (rDNAs, U1 snDNA, telomeric repeats) as probes, identified species-specific chromosome markers that have been compared with those of other species of the genus *Mugil*. Cytochrome c oxidase subunit I (COI) sequence analysis shows only 92–93% similarity with sequences previously deposited under this species name in GenBank, all of which were from the Atlantic Ocean. Phylogenetic reconstructions indicate the presence of three well-supported hospe mullet lineages whose molecular divergence is compatible with the presence of distinct species. Indeed, the first lineage includes samples from Ecuador, whereas the other two lineages include the Atlantic samples and correspond to *M. brevirostris* from Brazil and *Mugil* sp. R from Belize/Venezuela. Results here provided reiterate the pivotal importance of an integrative molecular and cytogenetic approach in the reconstruction of the relationships within Mugilidae.

## Introduction

Mullets is the popular name of fishes included in the Mugilidae, a species rich family that is the only representative of the order Mugiliformes. These fishes are distributed in several coastal aquatic habitats in tropical, subtropical and temperate regions of the world, where they are ecologically, recreationally and commercially important (Thomson, 1966). According to different authors (see González-Castro and Ghasemzadeh, 2016 and references herein), the family has approximately 26 genera, but Eschmeyer and Fong (2017) ascribe to Mugilidae 20 genera and 75 valid species.

In Mugilidae, most of the classical morphological characters used in species identification and/or systematics have poor diagnostic power and morphometric variability is limited (Schultz, 1946; Thomson, 1997; González-Castro, 2007; González-Castro and Ghasemzadeh, 2016). These characteristics are associated with the wide distribution of most of the species, which raises questions about their actual taxonomic status. Cytogenetic and molecular studies have provided important data for understanding the systematic relationships and evolutionary pathways among mullet species (Harrison et al., 2007; Sola et al., 2007; Durand et al., 2012; Durand and Borsa, 2015). These studies have also shown that it is necessary to use integrative approaches to study mugilids. Indeed, the use of repetitive sequences such as ribosomal genes (18S rDNA and 5S rDNA) as probes in FISH mapping has been shown to be a very informative cytotaxonomic tool in revealing different lineages/species within Mugilidae (Nirchio et al., 2007, 2017; Sola et al., 2007). On the other hand, the utility of molecular markers in this family to identify the species, better define the genera, and reconstruct their phylogenetic relationships, is well-represented by the huge amount of literature on this topic published in the last 15 years (see Rossi et al., 2016 for a review and Durand et al., 2017). In addition, molecular phylogenetic analyses have been used successfully in the investigation of chromosome evolution in some fish groups as those of the genus *Characidium* (Pansonato-Alves et al., 2014) and *Triportheus* (Yano et al., 2014), and in *Geophagus brasiliensis* (Alves-Silva and Dergam, 2015).

*Mugil*, which presently includes 16 valid species (Eschmeyer and Fong, 2017), is the most cytogenetically studied genus among the Mugilidae. Nine species have been investigated to date (see section “Discussion” and **Figure 6**). Nonetheless, the number of species is probably underestimated currently, as recent molecular data have indicated that there are different species complexes within this genus. For example, the cosmopolitan *M. cephalus* was found to be composed of 15 well supported mitochondrial lineages (Durand and Borsa, 2015), including the one sampled in the type-locality (Mediterranean Sea); six of these lineages have already been cytogenetically analyzed (Rossi et al., 1996, 2016). However, these lineages lack formal descriptions and species name attribution.

Very recently, Durand et al. (2017) reported the presence of two well-supported mitochondrial lineages in the hospe mullet “*Mugil hospes*,” a species that, according to Barletta and Dantas (2016), is distributed in the western Atlantic from Belize to Brazil and in the eastern Pacific from Mexico to Ecuador. The first molecular lineage includes sequences from Brazil and corresponds to the resurrected species *Mugil brevirostris*, which is distributed from the northern Brazilian coast (Amapá) to the southern Brazilian coast (Rio Grande do Sul) (Menezes et al., 2015); the second lineage is represented by haplotypes collected in the Gulf of Mexico (Belize/Venezuela) and was named *Mugil* sp. R. Samples from the eastern Pacific were not included in these analyses or in any other molecular study. The karyotype of the species remains undescribed.

In this research, specimens of *M. hospes* from Ecuador have been collected and their morphological characters accurately analyzed to make sure of the correct species identification. Cytogenetic and mitochondrial cytochrome c oxidase subunit I (COI) sequence analyses were performed aiming to (a) verify whether the original described *M. hospes* from the Pacific Ocean corresponds to one of the two lineages identified in the Atlantic Ocean or represents a third lineage, (b) estimate if the divergence among lineages is sufficient to attribute them to different species, (c) identify species-specific chromosome markers and add new comparative data that allow cytotaxonomic inferences on Mugilidae karyotype evolution.

## Materials and Methods

Fourteen specimens of *Mugil hospes* (four males, four females, six immature), were collected with a cast net from a reservoir that provides water to a shrimp pool located at Barbones, El Oro Province, Ecuador (3°09′14.0″ S 79°53′53.1″ W). Fishes were transported to the laboratory in sealed plastic bags (32′) containing two gallons of water, and the air in the bags was replaced with pure oxygen. All 14 individuals were used to prepare cell suspensions. A subsample of eight individuals was used for molecular and morphological analyses. Voucher specimens were deposited in the fish collection of the Laboratório de Biologia e Genética de Peixes (LBP), UNESP, Botucatu (São Paulo State, Brazil) (collection numbers LBP 23325) and Universidad Técnica de Machala (UTMACH-174-UTMACH-182; UTMACH-187; UTMACH-191-UTMACH-194). All experiments were conducted according to the Ethical Committee of Instituto de Biociências/UNESP/Botucatu, under protocol number 1057.

### Morphological Analysis

Each fish was measured. Measurements and counts were taken as described by Menezes et al. (2010). Mouth width and mouth depth were measured as described by Thomson (1997). Twenty morphometric characters (Supplementary Table 1) and nine meristic characters (Supplementary Table 2) were recorded for each fish.

### Molecular Analysis

Genomic DNA was extracted from muscle tissue that was preserved in 95% ethanol. DNA samples were obtained for eight specimens (one male, two female, five immature), according to procedures described by Aljanabi and Martínez (1997). A 655 bp fragment of the mitochondrial COI was amplified by PCR and sequenced using primers and protocols reported by Nirchio et al. (2017). DNA sequences were aligned using the software Clustal X (Thompson et al., 1997) and deposited in GenBank (Accession numbers: KY964500-KY964504). The basic local alignment search tool (BLAST^1^) was used to search for similar sequences to confirm species assignment.

For phylogenetic tree reconstruction, a subset of the COI sequences of *Mugil*, previously analyzed by Durand et al. (2017), was considered. Those sequences that showed greater than 90% similarity (i.e., the six sequences of *M. brevirostris* and the seven sequences of *Mugil* sp. R) were also included; *Agonostomus monticola* (Bancroft, 1834) (JQ060401) was used as an outgroup.

Three types of phylogenetic reconstructions were conducted: neighbour-joining (NJ), maximum-likelihood (ML) and Bayesian inference (BI) analyses. NJ and ML analyses (1000 bootstrap pseudoreplicates) were performed using MEGA7 (Kumar et al., 2016) and PhyML 3.0 (Guindon et al., 2010), respectively. Bayesian analyses were carried out as implemented in MrBayes 3.1.2 (Huelsenbeck and Ronquist, 2001). Two independent runs of four Markov chains, each for 1,000,000 generations were performed. ModelTest 3.7 (Posada and Crandall, 1998) and MrModelTest 2.3 (Nylander, 2008) were used to select, according to the Akaike information criterion, the evolutionary models that best fit the data set for the ML (GTR + I + G, with nst = 6, gamma shape = 4.682, and proportion of invariant sites = 0.637) and the BI (GTR + I + G) analyses, respectively. Genetic distances were calculated with MEGA7 using the Kimura-2-parameters substitution model (Kimura, 1980).

### Cytogenetic Analysis

Each fish received an intra-abdominal injection of 0.0125% colchicine (1.0 ml/100 g body weight) 50 min before being sacrificed by administering a numbing overdose of benzocaine (250 mg/L) as recommended by the Guidelines for the Euthanasia of Animals of the American Veterinary Medical Association (AVMA, 2013). Kidney cells were suspended, and chromosomes were prepared by following the conventional air-drying method, as described by Nirchio and Oliveira (2006). Classical staining techniques (Giemsa, Ag-staining, C-banding) and fluorescence *in situ* hybridization (FISH) were used to map ribosomal gene clusters (5S rDNA and 18S rDNA) and U1 snRNA gene clusters (U1 snRNA is a non-coding RNA that forms part of the spliceosome) (Nilsen, 2003). Telomeric probes were also applied. For the conventional karyotype, slides were stained for 20 min with 10% Giemsa in phosphate buffer at pH 6.88. Active nucleolus organizer regions (NORs) were revealed by silver (Ag) staining as described by Howell and Black (1980); this was performed after Giemsa staining (Rábová et al., 2015). C-banding was performed following the method of Sumner (1972).

The 5S rDNA, 18S rDNA, U1 snRNA genes and telomeric repeats were mapped onto chromosomes by FISH using the method described by Pinkel et al. (1986). Sequences of 5S rDNA, 18S rDNA, U1 snDNA and telomeric repeats were obtained by polymerase chain reaction (PCR) from the genome of *Hypsolebias flagellatus* and used as probes. The primers used for amplification were 5SA (5′-TCA ACC AAC CAC AAA GAC ATT GGC AC-3′) and 5SB (5′-TAG ACT TCT GGG TGG CCA AAG GAA TCA-3′) (Pendás et al., 1995), 18S6F (5′-CTC TTT CGA GGC CCT GTA AT-3′) and 18S6R (5′-CAG CTT TGC AAC CAT ACT CC-3′) (Utsunomia et al., 2016), U1F (5′-GCA GTC GAG ATT CCC ACA TT-3′) and U1R (5′-CTT ACC TGG CAG GGG AGA TA-3′) (Silva et al., 2015) and (TTAGGG)5 and (CCCTAA)5 (Ijdo et al., 1991). The 5S rDNA and telomeric probes were labeled with biotin-16-dUTP (2′-deoxyuridine 5′-triphosphate), and the 18S rDNA and U1 snRNA gene probes were labeled by including digoxigenin-11-dUTP in the PCR. Hybridization was detected with fluorescein-conjugated avidin (FITC, Sigma–Aldrich^2^) and anti-digoxigenin-rhodamine conjugate (Roche Applied Science^3^), respectively. Chromosomes were counterstained with 4,6-diamidino-2-phenylindole (DAPI), which was included in the Vectashield mounting medium (Vector Laboratories^4^).

Conventionally stained metaphase cells were photographed using a Motic B400, equipped with a Moticam 5000C digital camera using Motic Images Plus 2.0 ML software. FISH images were captured with an Olympus BX61 photomicroscope equipped with a DP70 digital camera using Image-Pro plus 6.0 software (Media Cybernetics). Images were merged and edited for optimization of brightness and contrast using Photoshop (Adobe Systems, Inc.) Version 2015.0.0.

## Results

### Meristic and Morphometric Characters

**FIGURE 1:**
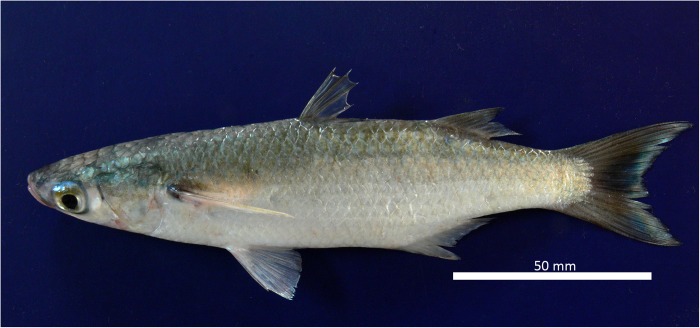
Specimen of *Mugil hospes* from Ecuador.

The fresh specimens were gray on the dorsal side and white/silver on the ventral side. The pelvic fins had a yellowish tone, and the base of each pectoral fin had a visible dark spot. The dorsal fins and caudal fins were dusky. The distal tips of the anterior rays of the second dorsal fin were slightly darker. The pelvic and anal fins were pale. The body was elongated, with a slightly pointed snout (see **Figure 1**). The origin of the first dorsal fin was midway between the tip of the snout and the base of the caudal fin. The second dorsal fin and anal fin were profusely covered with scales. One row of small teeth was visible on the upper and lower lips (viewed under the microscope). There were adipose eyelids and widely separated spiny-rayed dorsal fins with four spines in the first dorsal fin and one spine plus eight soft rays in the second dorsal fin (small specimen with nine soft rays). Pelvic fins were sub-abdominal with one spine and 5–6 branched soft rays (commonly I+5). Pectoral fins were long, reaching the level of the origin of the first dorsal fin or extending just beyond, with two spines (the first spine very small) and 11–13 soft rays (commonly 12 rays). The anal fin had three spines and nine soft rays (first spine very short, and hidden by overlying scales). There was a large pectoral axillary scale, with 37–38 scales in longitudinal series (commonly 38), 11–14 scales in an oblique row extending to the origin of the pelvic fin (commonly 13) and 13 scales in a transversal series, as well as 17–22 scales in a circum-peduncular series (commonly 19) (Supplementary Table 2).

### Molecular Analysis

BLAST was used to show that COI nucleotide sequences from GenBank have 92–93% similarity with specimens originally identified as *Mugil hospes*, and with *M. trichodon* and *Mugil* sp., which were all collected in the Atlantic Ocean (from Brazil and Belize). Similarity values with other *Mugil* species were all below 90%.

**FIGURE 2:**
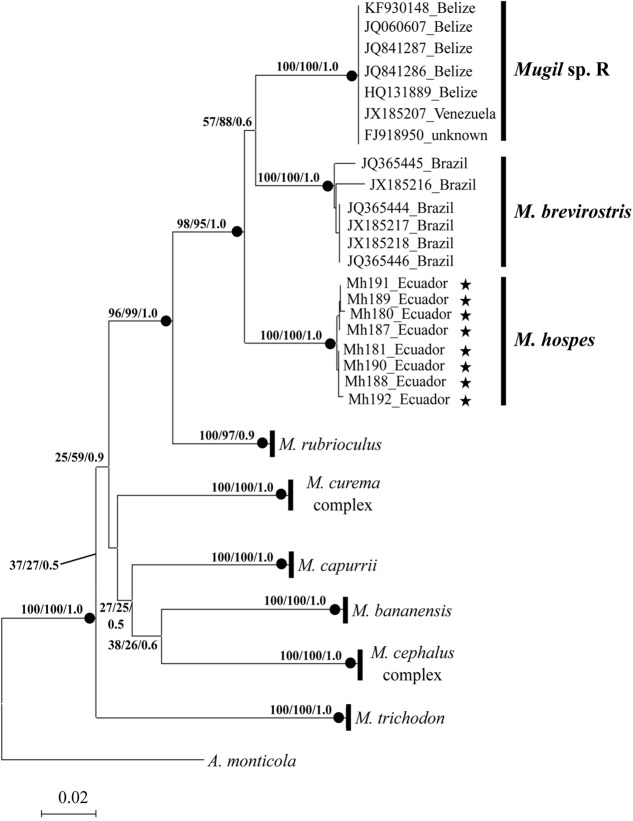
Neighbor-joining tree based on COI sequences. At each node bootstrap values (NJ and ML) and posterior probabilities (BI) are shown. Black dots at the nodes indicate bootstrap values > 70% (NJ and ML) and posterior probabilities > 0.9 (BI). Stars indicate sequences obtained in this study; the remaining sequences are from Durand et al. (2017).

The phylogenetic tree obtained by NJ, ML (lnL = -3276.75382), and BI (lnL = -3986.503923) analyses (**Figure 2**) shows three well-supported lineages of “*M. hospes*.” The first two correspond to the *M. brevirostris* (Brazil) and *Mugil* sp. R (Belize and Venezuela) lineages identified by Durand et al. (2017), whereas the third, referred to hereafter as *Mugil hospes* (see Discussion), includes all the sequences from Ecuador obtained in this study (**Figure 2**). The genetic distance is 0.077 between *M. hospes*/*M. brevirostris* and between *M. hospes*/*Mugil* sp. R, and 0.073 between *M. brevirostris/Mugil* sp. R.

### Cytogenetic Analysis

**FIGURE 3:**
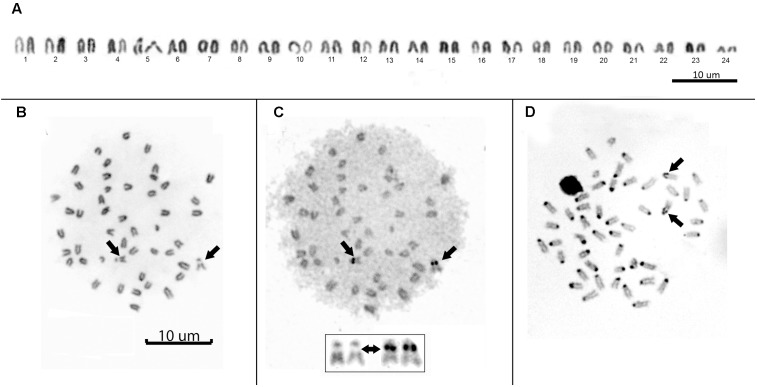
**(A)** Conventional Giemsa-stained karyotype of *Mugil hospes.* Sequential Giemsa **(B)** and AgNO_3_-staining **(C)** of the metaphase plate (inset shows NOR-bearing chromosomes); arrows indicate the NOR-bearing chromosome. **(D)** C-banded chromosomes; arrows indicate positive heterochromatic blocks on the interstitial secondary constriction of chromosome pair 5.

All individuals showed a diploid number of 2n = 48 and a karyotype composed entirely of uniformly decreasing acrocentric chromosomes. Thus, the fundamental arm number (NF) was 48. Only two pairs of homologous chromosomes can be identified with certainty: pair 5, due to a clear interstitial secondary constriction, and pair 24, which is distinctly small (**Figure 3A**). Sequential Giemsa-silver (Ag) nitrate staining enabled the identification of two actively transcribing NORs, interstitially located on the secondary constriction of chromosome pair 5 (**Figures 3B,C**). C-banding showed that constitutive heterochromatin is restricted to the centromeric regions of all chromosomes, and there is a pericentromeric heterochromatin block on the secondary constriction of chromosome pair 5 (**Figure 3D**).

**FIGURE 4:**
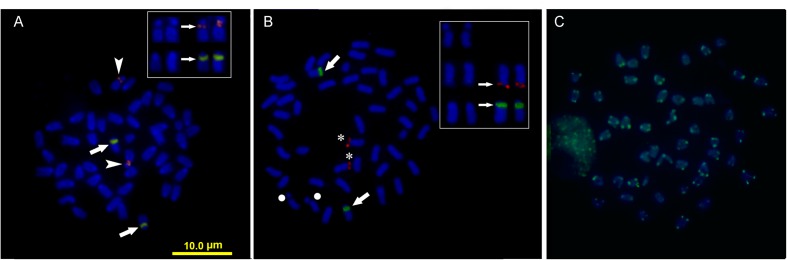
Somatic metaphase chromosomes of *Mugil hospes* assayed by FISH and counterstained with DAPI: **(A)** 5S rDNA (arrows) and 18S rDNA (arrowheads); **(B)** U1 snRNA (asterisks), 5S rDNA (arrows), chromosome pair 5 (circle), and **(C)** telomeric repeats. Enlargement of selected samples of chromosome pairs after DAPI staining (left) and FISH (right) are shown in the insets: **(A)** chromosome pairs 5 and 15, with probes showing 18S rDNA (above) and 5S rDNA (below) positive sites; **(B)** chromosome pair 5 (above), chromosome pair 10 (center) showing U1 snDNA positive sites and chromosome pair 15 (below) showing 5S rDNA.

**FIGURE 5:**
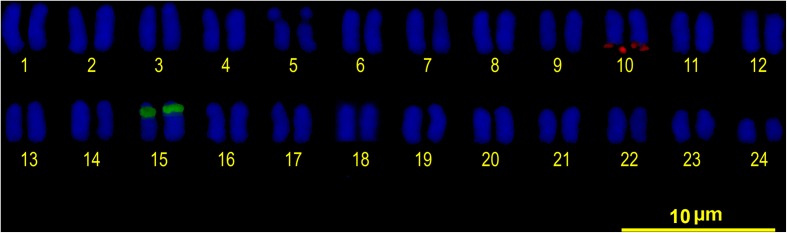
FISH karyotype. Interstitial secondary constriction corresponding to NOR (chromosome pair 5); 5S rDNA (chromosome pair 15) and U1 snDNA (chromosome pair 10) positive sites are evident.

Double FISH experiments using 5S and 18S rDNA as probes revealed two positive sites detected for each probe (**Figure 4A**), located on different chromosome pairs. The 18S rDNA positive sites correspond to the AgNO_3_ sites on the secondary constriction of chromosome pair 5. The 5S rDNA probes hybridized interstitially on a pair of medium-sized chromosomes. Double FISH using the U1 snDNA and 5S rDNA probes revealed positive U1 snDNA signals on the telomeric region of a pair of medium-sized chromosomes distinct from the 5S-rDNA-bearing chromosome pair (**Figure 4B**). When chromosomes treated by double FISH were sorted by decreasing size, it was possible to assign the 5S rDNA sites to pair 15 and the U1 snDNA signals to pair 10 (**Figure 5**). Telomeric repeats were located at both ends of each chromosome, although signal intensities varied between chromosomes (**Figure 4C**).

## Discussion

Meristic and morphometric data of samples from Ecuador agree with the original description of *M. hospes* (Jordan and Culver 1895 in Jordan, 1895): this species has pectoral fins whose tips reach and extend slightly past the vertical line passing through the origin of the first dorsal fin (with four spines). This morphological character is shared with *M. brevirostris*, which inhabits the opposite side of the Americas (i.e., the Atlantic coast).

Sequence analysis showed that samples of the hospe mullet from Ecuador are genetically very different from those collected in the Atlantic Ocean, all of which were originally identified with the same name *M. hospes*. Thus, in addition to the two lineages identified by Durand et al. (2017) in the Atlantic Ocean, *M. brevirostris* and *Mugil* sp. R, a third lineage is present in the Pacific Ocean. The genetic distances between the Pacific and the two Atlantic lineages are higher than the COI 2% threshold value that discriminates different species (Ward, 2009), and in the phylogenetic reconstruction, the three species form a monophyletic and well-supported clade. Cryptic species are defined as distinct evolutionary lineages not detectable with traditional taxonomic approaches, due to the absence of morphological differences (Avise, 2000; Mallet, 2010). In the last decade, barcoding methods based on COI sequences have made possible their identification in several marine and freshwater fish species (Ward et al., 2008; Lara et al., 2010; Puckridge et al., 2013; Mateussi et al., 2017; Okamoto et al., 2017; Ramirez et al., 2017; Shimabukuro-Dias et al., 2017). In Mugilidae evidences of cryptic species were inferred from mitochondrial tree topology, independent data from nuclear markers, and on the base of the geographic distribution of sister lineages (Durand and Borsa, 2015). Our results indicate that the genetic distances between the different hospe mullet are comparable to those reported among species within both the *M. cephalus* and *M. curema* species complexes (Durand et al., 2017), and the three lineages inhabits different geographic areas. Thus, we hypothesize that besides the *Mugil cephalus* and *Mugil curema* species complexes, there is an additional putative one, which should be identified as the *M. hospes* species complex; the name *M. hospes* should be kept by the Pacific samples, being Mazatlán (in the eastern Pacific) the species type-locality.

Cytogenetic analysis shows that the 48 acrocentric chromosome karyotype detected in *M. hospes* is consistent with the generally available data on diploid chromosome number and karyotype structure in Mugilidae (Sola et al., 2007; Rossi et al., 2016). This confirms that the only exception is represented by the mullets belonging to the *Mugil curema* species complex (Nirchio et al., 2017).

**FIGURE 6:**
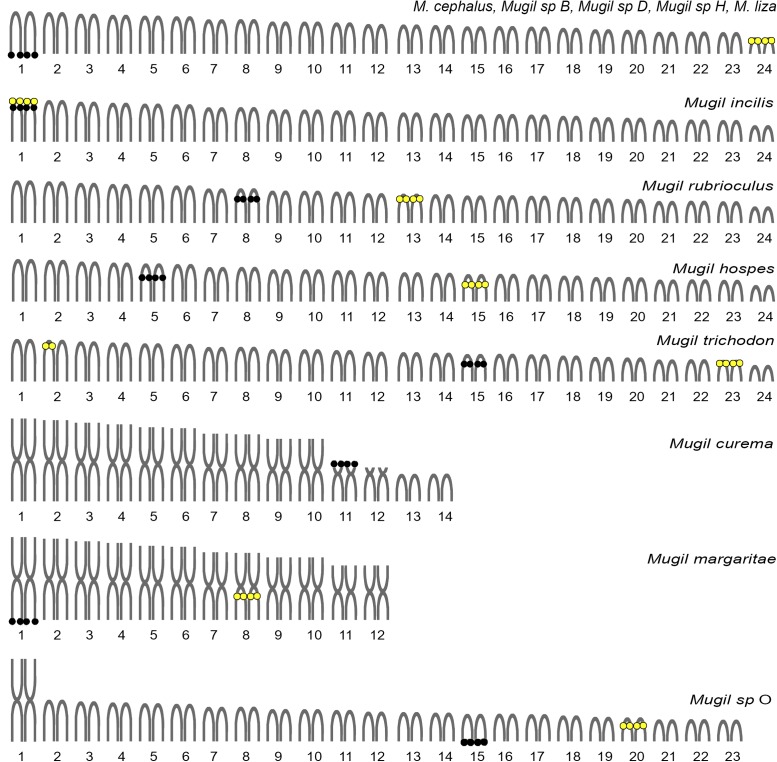
Idiograms of the karyotypes observed in *Mugil* species. Re-drawn and updated from Rossi et al. (2016). Black circles: major ribosomal gene locations. Yellow circles: minor ribosomal gene locations.

Apart from the number of chromosomes, many microstructural changes are evident looking at the variability in the locations of ribosomal genes in *Mugil*. For example, 5S rDNA cistrons are always localized to an interstitial position, although they are on different chromosomes in different species (**Figure 6**). The 18S rDNA cistrons seems to be more variable and can be found in the telomeric or interstitial regions of a long chromosome arm, or even on the short arms of different chromosomes. The variability in the localization of the major ribosomal genes could be attributable to their association with heterochromatinas that is observed in *Mugil cephalus*, *M. margaritae* (formerly *M. curema*), *M. rubrioculus*, *M. curema*, *M. liza*, *M. trichodon*, *M. incilis*, *Mugil* sp. O (Rossi et al., 1996, 2005; Nirchio et al., 2005a,b, 2007, 2017; Hett et al., 2011), and *M. hospes* (present study). Heterochromatin is known to evolve rapidly, and its composition, that includes highly repetitive simple sequences like satellite DNA and transposable elements, is often different even between closely related species. This characteristic might promote rearrangements of the associated genes and might play an important role in reproductive isolation between sister species (Hughes and Hawley, 2009).

Cytogenetic mapping of U1 snDNA probes in *M. hospes* showed the presence of a single U1 gene cluster, located in the terminal position of a chromosomal pair different from the 18S rDNA and 5S bearing chromosomes (**Figures 4B**, **5**). There are no data available on the chromosome mapping of these sequences in other Mugilidae; thus, it is not possible, at this stage, to compare our results with those of other species in the family. However, the mapping of these sequences, combined with other repetitive sequences in other *Mugil*, might allow the identification of other chromosome re-arrangement. The analysis of chromosome localization of these sequences is restricted to a few other fish species. In *Merluccius merluccius* (Merlucciidae), multiple interstitial U1 sites are present (García-Souto et al., 2015). In 19 species of cichlids (Cabral-de-Mello et al., 2012), these sites could be either interstitial or terminal on a single st/a chromosome pair, and represent good chromosomal markers that allow the detection of many microstructural chromosomal rearrangements. On the contrary, in five species of *Astyanax* (Characidae), there is a conserved pattern in the number of U1 sites per genome, and these sequences are frequently associated with 5S rDNA sequences (Silva et al., 2015).

Telomeric DNA repeat sequences were found at the very ends of chromosomes, as observed in 15 different orders of teleosts (Ocalewicz, 2013). In mugilids, telomeric repeats have been mapped in nine species (Gornung et al., 2004; Rossi et al., 2005; Nirchio et al., 2017) and were found also to be interspersed in NORs. Signal intensity variability between chromosomes, as observed in *M. hospes*, has been previously reported in other fishes (Rocco et al., 2002; Ocalewicz and Dobosz, 2009; Pomianowski et al., 2012), including *Mugil* species such as *M. cephalus* (Gornung et al., 2004), *M. liza* and *M. margaritae* (Rossi et al., 2005), and *Mugil* sp. O (Nirchio et al., 2017). This variability is probably due to differing copy numbers of these sequences in the different sites (Lansdorp et al., 1996).

## Conclusion

The data presented here confirm that a complex dynamic has played in the karyotype evolution of *Mugil*, and they reiterate the usefulness of cytogenetic and molecular data in the reconstruction of relationships among taxa within Mugilidae. Species of this family are usually characterized by morphological features that are “insufficient to describe its actual species diversity” (Durand and Borsa, 2015). The combined use of morphological, molecular and cytogenetic analysis is necessary in these fishes to avoid species misidentification and to reconcile the confused picture obtained by morphology-based taxonomy with molecular-based taxonomy. In the case of the *M. hospes* species complex, *Mugil* sp. R, which is distributed in the Caribbean Sea, still deserves a formal morphological description and specific name attribution. This species, along with the Brazilian species *M. brevirostris*, also requires a karyotype description. Thus, at this stage, it is not possible to determine whether this complex is characterized by karyotypes that differ in the total number and morphology of chromosomes, like the *M. curema* species complex (Nirchio et al., 2017), or whether it is characterized by karyotype homogeneity, like *M. cephalus* species complex (Rossi et al., 1996).

## Author Contributions

MN, CO, and AR designed the study. FP and VM conducted the lab work. MN, AR, and VM designed and conducted the analyses. All authors analyzed the results and wrote the manuscript.

## Conflict of Interest Statement

The authors declare that the research was conducted in the absence of any commercial or financial relationships that could be construed as a potential conflict of interest.

## Footnotes

1 https://blast.ncbi.nlm.nih.gov/Blast.cgi

2 www.sigma-aldrich.com

3 https://lifescience.roche.com/

4 https://vectorlabs.com/
