# Supplementary material for: Identification of a New Mullet Species Complex Based on an Integrative Molecular and Cytogenetic Investigation of Mugil hospes (Mugilidae: Mugiliformes)
Source: Front Genet. 2018 Feb 5;9:17. doi: 10.3389/fgene.2018.00017 (PMC5807406; doi:10.3389/fgene.2018.00017)
Supplement: Supplementary file 1 [file Table_1.PDF]

Supplementary Material

Identification of a New Mullet Species Complex Based on an Integrative Molecular and Cytogenetic Investigation of *Mugil hospes* (Mugilidae: Mugiliformes).

Mauro Nirchio, Fabilene Gomes Paim, Valentina Milana, Anna Rita Rossi, Claudio Oliveira*

***Correspondence:**
claudio@ibb.unesp.br

TABLE 1. Morphometric data of *Mugil hospes*. N=8.

Characters	Mean	Min	Max	SD	CV%
Standard Length (mm)	138.1	84.0	172.0	24.36	17.64
Percent of Standard length					
Body depth	23.15	20.40	24.60	1.34	5.78
Head length	26.64	25.80	27.20	0.40	1.50
Caudal peduncle depth	10.92	10.30	11.40	0.37	3.42
Pectoral fin Length	22.78	18.20	24.30	1.79	7.86
Pelvic fin Length	15.05	13.30	16.90	1.07	7.13
Axillary scale Length	9.47	4.50	13.10	2.34	24.76
Snout to Dorsal I fin	50.80	49.60	52.70	1.12	2.20
Snout to Dorsal II fin	74.42	71.90	75.90	1.24	1.67
Snout to Pectoral fin origin	28.82	27.60	29.80	0.77	2.67
Snout to Pelvic fin origin	39.80	38.60	40.70	0.60	1.51
Snout to Anal fin origin	67.94	64.30	74.70	3.20	4.70
Percent of Head length					
Head depth	65.20	60.86	69.89	3.56	5.46
Head width	58.30	53.13	61.79	3.18	5.45
Upper jaw Length	27.90	24.74	29.81	1.57	5.54
Mouth depth	30.03	27.11	32.76	1.93	6.43
Horizontal orbital diameter	25.35	20.77	30.51	2.82	11.12
Snout Length	28.09	25.78	29.88	1.32	4.70
Interorbital distance	40.61	34.64	49.99	4.10	10.11
