# Supplementary material for: Identification of a New Mullet Species Complex Based on an Integrative Molecular and Cytogenetic Investigation of Mugil hospes (Mugilidae: Mugiliformes)
Source: Front Genet. 2018 Feb 5;9:17. doi: 10.3389/fgene.2018.00017 (PMC5807406; doi:10.3389/fgene.2018.00017)
Supplement: Supplementary file 2 [file Table_2.doc]

**Supplementary Table 2.** Meristic data of *Mugil hospes*. N=8.

|  | Min | Max | Mode |
| --- | --- | --- | --- |
| Number of scales in longitudinal row | 37 | 38 | 38 |
| Number of scales in oblique row until the origin of the pelvic fin | 11 | 14 | 13 |
| Number of transversal scales | 13 | 13 | 13 |
| Number of circumpedular scales | 17 | 22 | 19 |
| First dorsal fin | IV | IV | IV |
| Second dorsal fin | I+8 | I+8 | I+8, very small specimens  with 9 rays |
| Pectoral fin | II+11 | II+13 | II+12 |
| Pelvic fin | I+5 | I+6 | I+5 |
| Anal fin | III+9 | III+9 | III+9 |
